# MicroRNA‐194 protects against chronic hepatitis B‐related liver damage by promoting hepatocyte growth via ACVR2B

**DOI:** 10.1111/jcmm.13714

**Published:** 2018-07-25

**Authors:** Xue Gao, Pan Zhao, Jie Hu, Hongguang Zhu, Jiming Zhang, Zhongwen Zhou, Jingmin Zhao, Feng Tang

**Affiliations:** ^1^ Department of Pathology 302 Hospital Beijing China; ^2^ Clinical Trial Center Beijing 302 Hospital Beijing China; ^3^ Liver Surgery Department Zhongshan Hospital Fudan University Shanghai China; ^4^ Liver Cancer Institute Fudan University Shanghai China; ^5^ Department of Pathology Shanghai Medical College Fudan University Shanghai China; ^6^ Department of Pathology Huashan Hospital Fudan University Shanghai China; ^7^ Department of Infectious Diseases Huashan Hospital Fudan University Shanghai China

**Keywords:** ACVR2B, chronic hepatitis B, liver regeneration, microRNAs, miR‐194

## Abstract

Persistent infection with the hepatitis B virus leads to liver cirrhosis and hepatocellular carcinoma. MicroRNAs (miRNAs) play an important role in a variety of biological processes; however, the role of miRNAs in chronic hepatitis B (CHB)‐induced liver damage remains poorly understood. Here, we investigated the role of miRNAs in CHB‐related liver damage. Microarray analysis of the expression of miRNAs in 22 CHB patients and 33 healthy individuals identified miR‐194 as one of six differentially expressed miRNAs. miR‐194 was up‐regulated in correlation with increased liver damage in the plasma or liver tissues of CHB patients. In mice subjected to 2/3 partial hepatectomy, miR‐194 was up‐regulated in liver tissues in correlation with hepatocyte growth and in parallel with the down‐regulation of the activin receptor ACVR2B. Overexpression of miR‐194 in human liver HL7702 cells down‐regulated ACVR2B mRNA and protein expression, promoted cell proliferation, acceleratedG1 to S cell cycle transition, and inhibited apoptosis, whereas knockdown of miR‐194 had the opposite effects. Luciferase reporter assays confirmed that ACVR2B is a direct target of miR‐194, and overexpression of ACVR2B significantly repressed cell proliferation and G1 to S phase transition and induced cell apoptosis. ACVR2B overexpression abolished the effect of miR‐194, indicating that miR‐194 promotes hepatocyte proliferation and inhibits apoptosis by down‐regulating ACVR2B. Taken together, these results indicate that miR‐194 plays a crucial role in hepatocyte proliferation and liver regeneration by targeting ACVR2B and may represent a novel therapeutic target for the treatment of CHB‐related liver damage.

## 1 INTRODUCTION

Infection with the hepatitis B virus (HBV), which affects more than 300 million people worldwide, can lead to acute or chronic infection, liver cirrhosis and primary liver cancer.^1^, ^2^ Although the percentage of patients progressing to liver disease or liver cancer is low, the mortality of these diseases is serious, with approximately 600 000 HBV‐related deaths estimated annually and 73% of liver cancer deaths attributed to hepatitis viruses.^3^ Approximately 8%‐20% of patients with chronic hepatitis B (CHB) develop liver cirrhosis, and among these, the risk of hepatocellular carcinoma (HCC) is 2%‐5%.^4^ HBV infection is responsible for 50%‐80% of HCC cases. Chronic liver injury leads to liver cell death and regeneration of parenchymal cells.^5^ However, persistent liver injury can lead to failure of regeneration and the replacement of hepatocytes by extracellular matrix (ECM) mainly derived from hepatic stellate cells.^6^, ^7^ Therefore, a better understanding of the mechanisms underlying HBV‐related liver injury and the development of early detection methods to prevent progression to cirrhosis and HCC are essential for the prevention and treatment of liver diseases.

MicroRNAs (miRNAs) are small endogenous non‐coding RNAs that modulate gene expression by binding to the 3′‐untranslated region (3′‐UTR) of target mRNAs to inhibit translation or induce mRNA degradation.^8^ miRNAs are involved in many physiological processes including cell proliferation, differentiation and apoptosis, and aberrant expression of miRNAs is associated with several diseases.^9^ Certain miRNAs play a role in the maintenance of hepatocyte function, such as miR‐122a, which promotes the replication of the hepatitis C virus in hepatocytes, and miR‐150 and miR‐194, which inhibit hepatic stellate cell activation and extracellular matrix production associated with liver fibrosis.^10^, ^11^, ^12^ miRNAs such as miR‐122 and miR‐192 are overexpressed in the livers of mice exposed to drug‐induced liver injury in a pattern mimicking that of serum aminotransferase levels and the histopathology of liver degeneration. The same miRNAs are overexpressed in humans with acetaminophen‐induced acute liver injury, suggesting their value as markers of liver damage.^13^, ^14^

Activins are secreted polypeptides that belong to the transforming growth factor beta (TGFβ) superfamily, which plays a role in the regulation of cell proliferation, apoptosis, inflammation and differentiation in many cell types and organs including the liver.^15^ Activins are homo‐ or heterodimers of different β subunits that either dimerize with another β subunit to form activins or dimerize with a single α subunit to form inhibins.^15^, ^16^ Activin A, which functions as an inhibitor of liver growth, signals through two types of transmembrane serine threonine kinase receptors, binding first to type II activin receptors, which recruit and phosphorylate type I receptors.^17^, ^18^ There are two type II receptors, ActR‐II or ACVR22 and ActR‐IIB or ACVR2B. Similar to other TGFβ family receptors, activin A receptors recruit Smads 2 and 3 to transduce signals to the nucleus, although the existence of Smad‐independent signalling pathways has been suggested.^15^, ^19^, ^20^

In a previous study, we identified a plasma miRNA panel for the diagnosis of hepatitis B‐related HCC in a cohort of 934 patients with CHB, cirrhosis and HBV‐related HCC using microarray technology.^21^ In this study, we expanded this work by exploring the role and mechanisms of miR‐194, one of six differentially expressed miRNAs in CHB, and its target ACVR2B in liver injury and regeneration associated with CHB.

## 2 MATERIALS AND METHODS

### 2.1 Microarray analysis and quantitative reverse‐transcriptase PCR (qRT‐PCR) validation

This study was approved by the local institutional review boards and informed consent was obtained from all study participants. Between August 2008 and June 2012, 231 participants (91 healthy and 140 CHB) who met the eligibility criteria (Table S1) were recruited from Shanghai Zhongshan Hospital, Huashan Hospital and Public Health Clinical Center. The 231 blood samples were allocated to two phases in chronological order (Figure S1A). There was no sample overlapping among the two phases. Table S2 shows the clinical characteristics of the study subjects. We used human miRNA microarray 2.0 from Agilent Technologies (Santa Clara, CA) to identify candidate miRNAs in 55 plasma samples (33 healthy and 22 CHB) as described in our previous study.^21^ The details of microarray hybridization are described in Appendix S1.

For testing of candidate miRNAs identified on microarrays, qRT‐PCR was performed using Taqman microRNA assays (Applied Biosystems, FosterCity, CA, USA). The assay was performed on plasma samples of 118 CHB patients and 58 healthy individuals for 9 candidate miRNAs (miR‐122, miR‐122*, miR‐148a, miR‐192, miR‐194, miR‐23b, miR‐215, miR‐27b, and miR‐29b) that met the defined criteria (Figure S1B). The expression levels of miRNAs were normalized to miR‐1228 levels 21, 22 and calculated using the 2^−▵Ct^ method. All assays were performed in triplicate.

### 2.2 Histological examination of liver biopsy specimens

Sixty‐six CHB patients suspected to have HBV‐related liver damage underwent liver biopsy. The biopsy specimens were fixed with 10% formalin, routinely embedded in paraffin, and tissue sections were stained with haematoxylin and eosin (H&E). The H&E sections with at least six portal tracts were blindly and independently assessed by three experienced pathologists, and the severity of chronic liver damage was evaluated according to the Scheuer classification system as follows^23^: G0, none; G1, inflammation but no necrosis; G2, focal necrosis or acidophil bodies; G3, severe focal cell damage; and G4, damage including bridging necrosis.

### 2.3 Animals and operative procedure

Healthy male C57BL/6J mice (n = 36, purchased from Shanghai Experimental Animal Center, Shanghai, China), aged 8‐10 weeks were housed (2‐4 mice per cage) in an animal room under specific pathogen‐free conditions with 22 ± 2.0°C indoor temperature and a 12‐hours light/dark cycle, and had free access to water and standard chow. All animal experiments were performed in a humane manner in accordance with the Institutional Animal Care Instructions. The study protocol was approved by the Animal Ethics Committee of Fudan University.Two‐thirds of the liver was surgically removed under isoflurane anaesthesia as previously described.^24^ Mice were killed and the remnant liver tissues were collected at 1, 3, 5, 7, and 14 days after surgery. One part was frozen in liquid nitrogen and stored at −80°C; the other part was fixed in 4% paraformaldehyde. Liver and body weight were recorded.

### 2.4 Immunohistochemical staining

The fixed liver tissues were embedded and 5 μm thick sections were prepared. The slides were stained with anti‐Ki‐67, anti‐proliferating cell nuclear antigen (PCNA), anti‐cyclin D1 and anti‐ACVR2B (Abcam, Cambridge, UK) and visualized with DAB (Sigma, St. Louis, MO, USA) followed by counterstaining with H&E (Sigma).

### 2.5 Cell culture and treatment

Human HL7702 normal liver cells were maintained in RPMI‐1640 medium (Gibco, USA) supplemented with 10% foetal bovine serum (Gibco) and 1% penicillin‐streptomycin (Gibco) at 37°C in 5% CO_2_. For overexpression of miR‐194, a miR‐194 mimic (Ambion, Austin, USA) was added to complexes at a final concentration of 60 nmol/L. For miR‐194 knockdown, antisense oligonucleotides (Ambion) were added to complexes at a final concentration of 100 nmol/L. The mirVana™ miRNA mimic negative control (Ambion) was applied as a control. For ACVR2B overexpression, ACVR2B cDNA was amplified and cloned into the pcDNA3.0 vector (Clontech, Palo Alto, USA). Transfections were performed using a Lipofectamine 2000 kit (Life Technologies, Carlsbad, USA) according to the manufacturer's instructions. Transfected cells were harvested at 24 or 48 hours.

### 2.6 Luciferase assay

The 3′‐UTR of human ACVR2B was cloned downstream of the luciferase gene into the luciferase reporter vector pGL3 (Promega, WI, USA). A mutant 3′‐UTR of ACVR2B was constructed using the Quick Change II XL Site‐Directed Mutagenesis Kit (Agilent Technologies, USA). At 2 hours after seeding into 6‐well plates, cells were cotransfected with the indicated luciferase reporter constructs and miR‐194 mimic, anti‐miR‐194, or negative control (Ambion) using Lipofectamine 2000 (Life Technologies) according to the manufacturer's instructions. Cells were harvested at 24 hours after transfection, and luciferase activity was measured using a dual‐luciferase reporter assay system (Promega).

### 2.7 qRT‐PCR

Total RNA was isolated from prepared mouse liver samples or transfected cells using the Trizol reagent (Invitrogen, CA, USA). cDNA was synthesized using Taqman RT reagents (Applied Biosystems, CA, USA) following the manufacturer's protocol. qRT‐PCR was performed using a standard SYBR green PCR kit (Qiagen, Hilden,Germany), and PCR‐specific amplification was performed using the Applied Biosystems (ABI7300) real‐time PCR machine. The relative expression of target genes was calculated with the 2^−▵▵Ct^ method. miR‐194 expression was normalized to U6 and ACVR2B to β‐actin. Primers are listed as follows: miR‐194, 5′‐CGA TCT CTC ATG TAA CAG CAA CTC‐3′ (F), 5′‐TAT GGT TGT TCT CGT CTC TGT GTC‐3′ (R); U6, 5′‐ATT GGA ACG ATA CAG AGA AGA TT‐3′ (F), 5′‐GGA ACG CTT CAC GAA TTT G‐3′ (R); ACVR2B, 5′‐GGC TGC TGG CTA GAT GAC TT‐3′(F); 5′‐AAG CGT TCG TTG CAG AAG TT‐3′(R); β‐actin, 5′‐TAT GCT CTC CCT CAC GCC A‐3′(F); 5′‐TTT ACG GAT GTC AAC GTC ACA C ‐3′(R).

### 2.8 Western blot analysis

Tissues and cells were lysed in RIPA lysis buffer (Santa Cruz Biotechnology, CA, USA). The lysates were sonicated and centrifuged at 11 750 ***g*** at 4°C for 10 minutes. Equal amounts of protein were separated using 10% sodium dodecyl sulphate‐polyacrylamide gel electrophoresis and transferred to nitrocellulose membranes. For immunodetection, membranes were incubated with primary antibodies against ACVR2B, p53, p‐Smad2, p‐Smad3, and Smad2/3 (Abcam). The immunoblots were developed using horseradish peroxidase (HRP)‐coupled secondary antibodies (Abcam) followed by detection with an ECL kit (Pierce Biotechnology, Rockford, USA). β‐actin was used as a control.

### 2.9 MTT cell proliferation assay

Cell proliferation rates were measured using 3‐(4,5‐dimethylthiazol‐2‐yl)‐2,5‐diphenyltetrazolium bromide (MTT) assays. At 24 hours after transfection, cells were cultured at a density of 1 × 10^4^ cells/well in a 96‐well plate for 24, 48, 72, and 96 hours. The cells were stained with 5% MTT (Sigma, 5 mg/mL) at the indicated time points followed by solubilization of the crystals in DMSO for 20 minutes at room temperature. Absorbance was measured at 570 nm.

### 2.10 Flow cytometry analysis

Cells were cultured in 96‐well plates and treated with the indicated oligonucleotides and/or vectors. After 48 hours, cells were collected, fixed with 70% ethanol for 30 minutes, and then washed with ice‐cold PBS for cell cycle analysis. The cell pellets were resuspended in RNase‐containing (1:100 dilution; Sigma) PBS buffer on ice. Finally, the cells were stained with propidium iodide (PI; 0.05 mg/mL, Sigma) and then analysed by flow cytometry and the CellQuest software (BD Bioscience, San Jose, CA). For apoptosis rate analysis, cells were evaluated using a FITC‐conjugated Annexin‐V and PI kit according to the manufacturer's instructions and analysed using a flow cytometer (BD Bioscience) and FlowJo software.

### 2.11 Statistical analysis

For microarray analysis, the Mann–Whitney unpaired test with Benjamini‐Hochberg correction was used for the comparison between CHB patients and healthy individuals. Hierarchical clustering analysis was performed with GeneSpring GX10 software (Agilent Technologies, Santa Clara, CA). For the data obtained by qRT‐PCR, the Mann–Whitney unpaired test was used for pairwise comparisons (CHB vs. healthy and CHB G0‐1 vs G2‐4).

The data shown in animal and cell experiments are presented as the mean ± standard deviation (SD). All *P* values were two‐sided and a *P*‐value of <.05 was considered statistically significant. The Student's *t* test was used to compare continuous variables between two groups.

## 3 RESULTS

### 3.1 miR‐194 is up‐regulated in correlation with increased liver damage in CHB patients

Hierarchical clustering analysis of microarray data showed that 33/33 healthy individuals and 21/22 CHB patients were correctly classified using differentially expressed miRNAs (Figure S1C). Candidate miRNAs were selected from the microarray platform using two eligibility criteria: (i) corrected *P* ≤ .0001 with fold change > 10, and (ii) normalized signal value ≥30 in the plasma of CHB patients. Nine candidate miRNAs met the criteria and were validated in an independent cohort of 176 plasma samples including 118 CHB patients and 58 healthy individuals using qRT‐PCR. Of the nine candidates, seven plasma miRNAs (miR‐122, miR‐148a, miR‐192, miR‐194, miR‐215, miR‐27b, and miR‐29b) showed significantly higher expression in CHB patients than in healthy individuals (Table S3).

**Figure 1:**
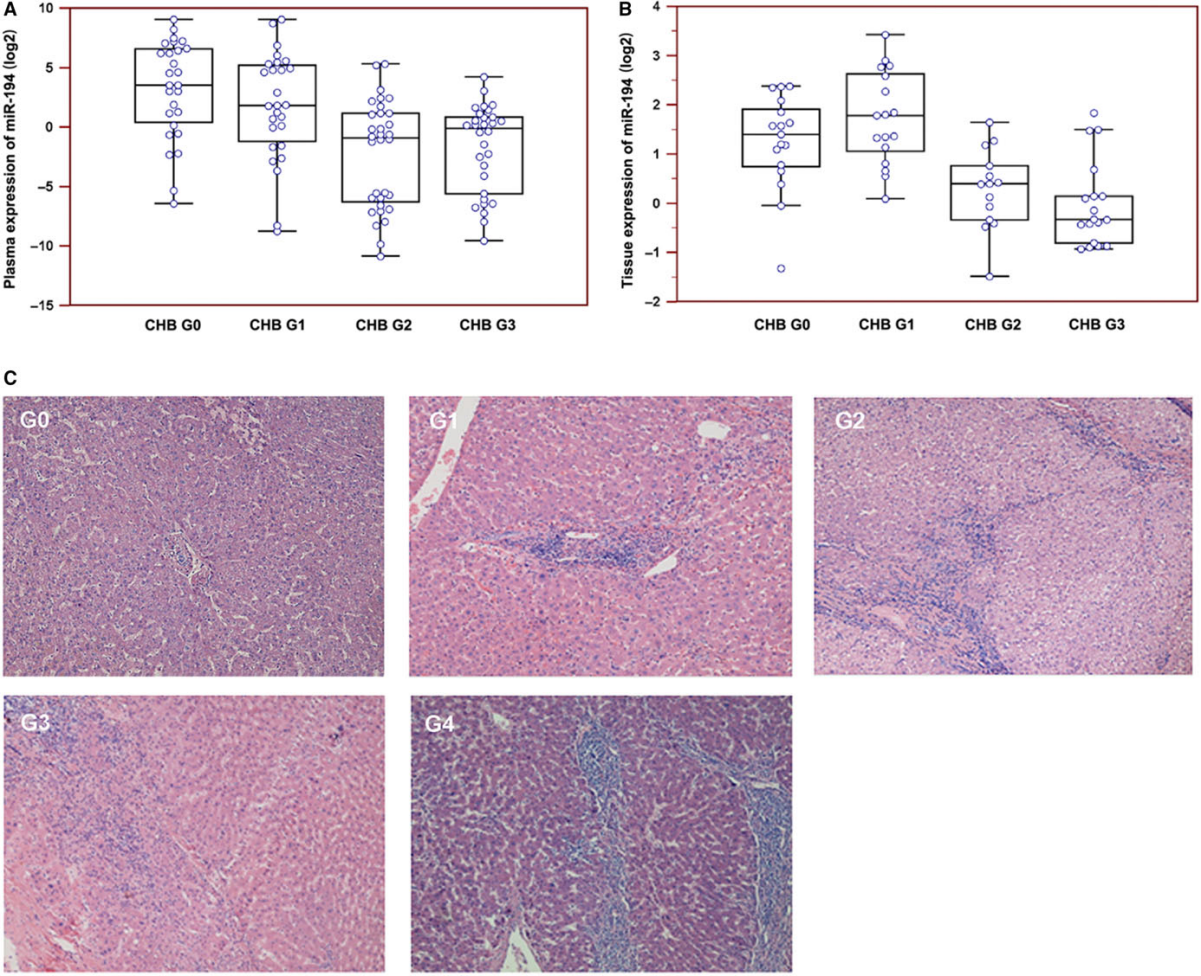
Involvement of miR‐194 in liver injury and regeneration in chronic hepatitis B (CHB) patients. A and B, The expression of miR‐194 in the plasma and liver tissues of CHB patients was detected by qRT‐PCR. Expression was shown in terms of ΔCt on a log_2_ scale with lower numbers indicating increasing expression. C, Histological liver damage was detected by H&E staining

According to the Scheuer Classification System of histological grades of liver damage, G0 and G1 patients had no necrosis, whereas G2, G3, and G4 patients showed necrosis. Therefore, CHB patients were divided into two groups, G0‐1 without significant liver damage and G2‐4 with significant liver damage. Analysis of the 118 CHB patients who underwent liver biopsy showed higher expression levels of miR‐122, miR‐148a, miR‐192, miR‐194, miR‐215, and miR‐27b in CHB patients with G2‐4 than in CHB patients with G0‐1 (fold changes: 3.1, 3.2, 2.6, 2.6, 3.3, and 1.9, respectively; Table 1), whereas there was no statistically significant difference between G0 and G1, or G2 and G3/4. Further analysis of miR‐194 expression in plasma and tissues of CHB patients with different histological grades of liver damage also showed higher expression levels in the G2 and G3 groups than in the G0 and G1 groups (Figure 1A and B). Figure 1C shows representative H&E stained images of histological liver damage in CHB patients in the different groups.

**Table 1:** Expression profiles of candidate miRNAs in CHB Patients

	Plasma		Tissue	
	*P*‐value	Fold change	*P*‐value	Fold change
Liver damage grade				
G2‐4 vs G0‐G1				
miR‐122	<.0001	5.9	<.0001	3.1
miR‐148a	<.0001	5.1	<.0001	3.2
miR‐192	<.0001	4.7	<.0001	2.6
**miR‐194**	**<.0001**	**22.6**	**<.0001**	**2.6**
miR‐215	<.0001	6.2	<.0001	3.3
miR‐27b	<.0001	1.3	<.0001	1.9

The miRNA selected for study is shown in bold.

### 3.2 miR‐194 is induced in response to partial hepatectomy

**Figure 2:**
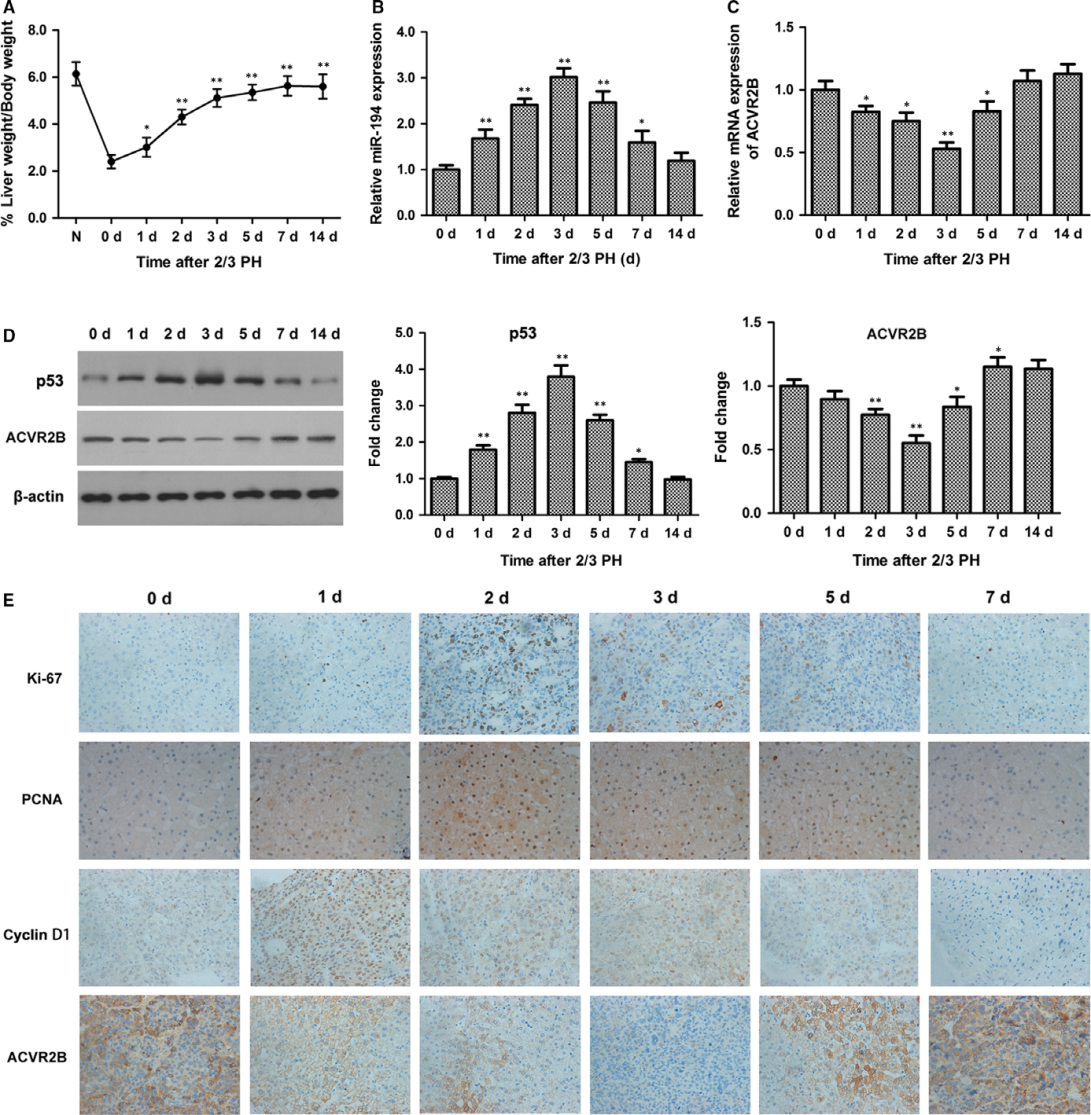
miR‐194 was up‐regulated in a mouse model of partial hepatectomy (PH). A, Recovery of functional liver mass after PH (normalized liver to body weight ratio). Data represent the mean ± SEM with n = 6 per group. B, The expression of miR‐194 in mouse liver tissues was detected by qRT‐PCR. C and D, The mRNA and protein expression levels of ACVR2B were determined by qRT‐PCR and Western blotting, respectively. *P < .05, **P < .01. (E) Liver immunohistochemical staining for Ki‐67, PCNA, cyclin D1, and ACVR2B. Original magnification, 200 ×

To determine the impact of miR‐194 on liver hepatocyte growth, mice were subjected to 2/3 partial hepatectomy (PH). As shown in Figure 2A, 2/3 PH promoted hepatocyte growth at a fast rate from 0 to 3 days, and then slowly from 3 to 7 days. miR‐194 was significantly up‐regulated on days 0‐3 and then restored on days 5‐14 (Figure 2B), whereas ACVR2B mRNA (Figure 2C) and protein (Figure 2D and E) showed the reverse pattern of expression in response to2/3 PH. Because miR‐194 is a target of p53, the expression of p53 was determined by Western blot analysis and was consistent with miR‐194 expression. Immunohistochemical staining showed that increasing numbers of hepatocytes entered the G1 phase of the cell cycle (Cyclin D1, brown) and progressed into S phase (PCNA and Ki‐67, both brown) from 0 to 3 days, and then declined to normal on days 5‐14 (Figure 2E). Taken together, these results implicate miR‐194 and its target ACVR2B in the response of hepatocytes to injury.

### 3.3 miR‐194 regulates ACVR2B at the post‐transcriptional level

**Figure 3:**
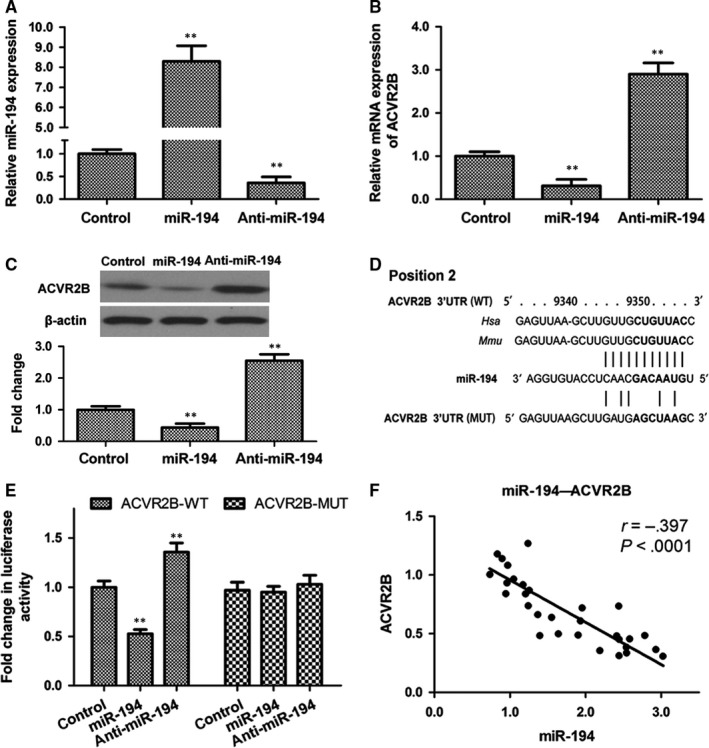
miR‐194 binds to and directly targets ACVR2B. A, qRT‐PCR analysis of miR‐194 expression in the human normal liver cell line HL7702 transfected with miR‐194, anti‐miR‐194, or negative control. B and C, The mRNA and protein expression levels of ACVR2B were determined by qRT‐PCR and Western blotting, respectively. D, Wild‐type or mutant ACVR2B 3′‐UTR‐binding sites for miR‐194. E, Relative luciferase activity of HL7702 cells after cotransfection with wild‐type (WT) or mutant (MUT) ACVR2B 3′‐UTR reporter constructs along with miR‐194, anti‐miR‐194, or negative control. N = 3. **P < .01. F, Correlation analysis of the mRNA expression of miR‐194 and ACVR2B in mouse liver tissues

To determine the effect of miR‐194 on hepatocyte growth, miR‐194 was overexpressed or silenced in the normal human liver cell line HL7702 by transfection with miR‐194 mimic or anti‐miR‐194. The efficacy of transfection was assessed by qRT‐PCR (Figure 3A). miR‐194 overexpression resulted in a significant down‐regulation of ACVR2B mRNA and protein, whereas miR‐194 knockdown had the opposite effect (Figure 3B and C). Luciferase reporter assays were performed to determine whether the two 3′‐UTR regions of ACVR2B (Figure S2A) were indeed functional target regions of miR‐194. The results showed that only position 2 showed decreased luciferase activity (Figure S2B). These results were confirmed using the dual‐luciferase reporter assay system, which showed that miR‐194 overexpression and silencing significantly inhibited and promoted luciferase reporter activity, respectively, in the wild‐type but not the mutant construct (Figure 3D and E). Correlation analysis showed a significant negative correlation between the mRNA expression of miR‐194 and ACVR2B in mouse liver tissues (Figure 3F).

### 3.4 miR‐194 promotes cell proliferation and inhibits apoptosis in human HL7702 liver cells

**Figure 4:**
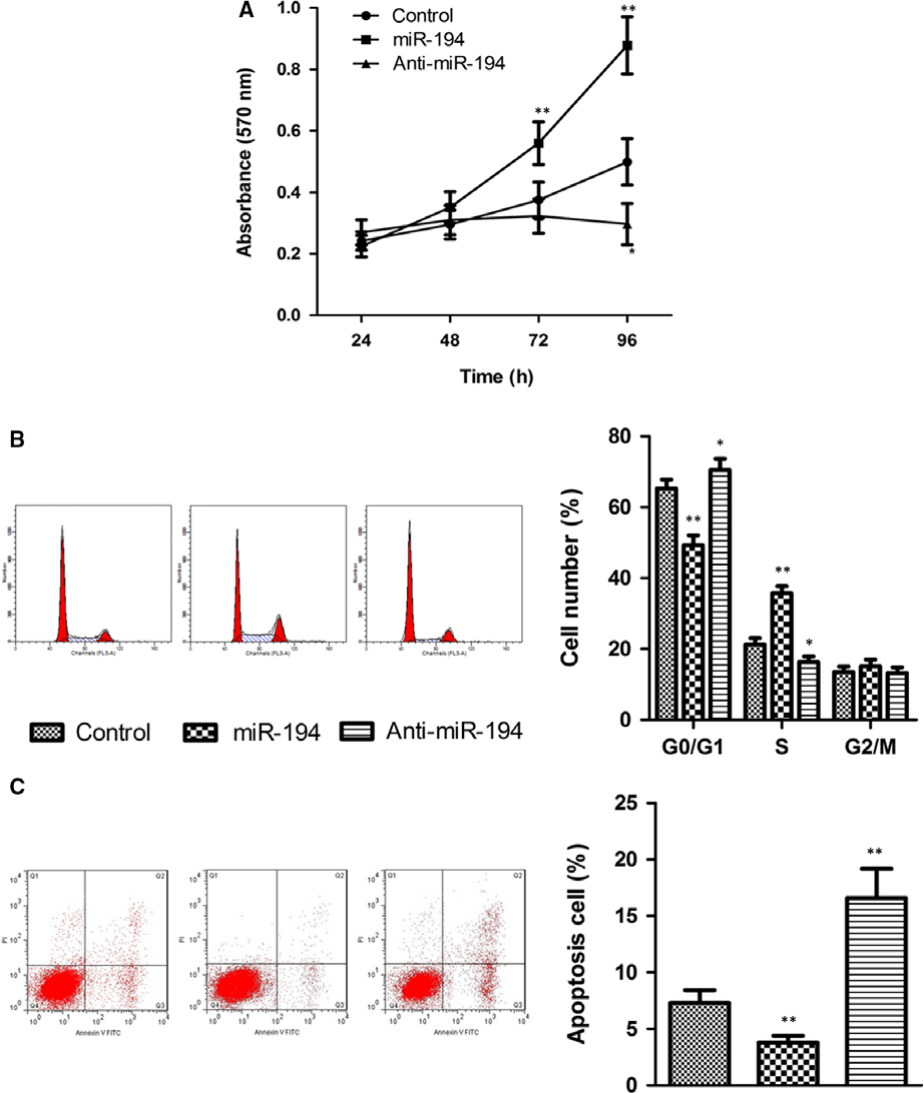
miR‐194 promotes cell proliferation and inhibits cell apoptosis in hepatic cells. A, Effect of miR‐194 on HL7702 cell proliferation. The human normal liver cell line HL7702 was transfected with miR‐194, anti‐miR‐194, or negative control and cell proliferation was assessed using the MTT assay. B and C, Flow cytometry analysis of the effects of miR‐194 on cell cycle distribution and cell apoptosis. n = 3. *P < .05, **P < .01

The effect of miR‐194 on hepatocyte growth was determined in the normal human liver cell line HL7702. Transfection of cells with miR‐194 mimics or anti‐miR‐194 and assessment of cell viability with the MTT assay showed that miR‐194 overexpression promoted HL7702 cell proliferation in a time‐dependent manner, whereas miR‐194 inhibition had the opposite effect (Figure 4A). Flow cytometry showed that miR‐194 overexpression accelerated cell cycle G1 to S phase transition (Figure 4B) and significantly inhibited cell apoptosis (Figure 4C), whereas miR‐194 knockdown had the opposite effects.

### 3.5 miR‐194 promotes hepatocyte proliferation and inhibits apoptosis by targeting ACVR2B

**Figure 5:**
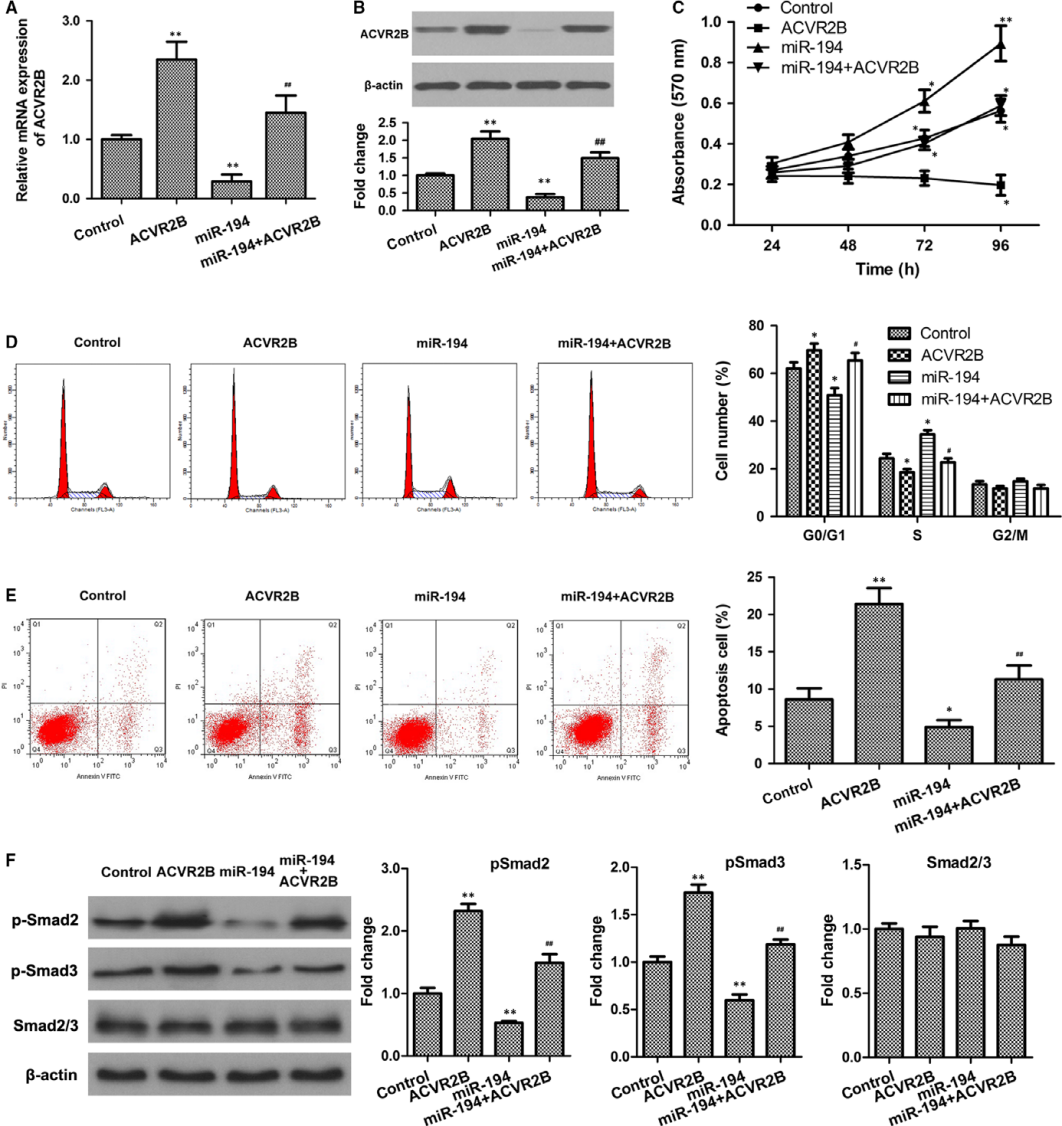
ACVR2B reversed the effect of miR‐194 on hepatic cell growth and apoptosis. A and B, The mRNA and protein expression levels of ACVR2B were determined by qRT‐PCR and western blotting, respectively. C, Cell proliferation was assessed using the MTT assay in HL7702 cells transduced with ACVR2B, miR‐194, miR‐194 + ACVR2B, or negative control. D and E, Cell cycle distribution and cell apoptosis were analysed by flow cytometry. F, Expression levels of phosphorylated Smad2/3 were determined by western blotting. n = 3. *P < .05, **P < .01 vs control; #*P* < .05, ##*P* < .01 vs miR‐194

The mechanism underlying the effect of miR‐194 on hepatocyte growth was further assessed in HL7702 cells overexpressing ACVR2B, miR‐194, or miR‐194 and ACVR2B. The efficacy of miR‐194 and ACVR2B overexpression was determined by qRT‐PCR and western blotting (Figure 5A and B). The results of the MTT assay and flow cytometry showed that ACVR2B overexpression significantly repressed cell proliferation and cell cycle G1‐S phase transition, and significantly induced cell apoptosis (Figure 5C‐E). In addition, ectopic expression of ACVR2Breversed the effect of miR‐194 on promoting cell proliferation and inhibiting apoptosis, indicating that miR‐194 promotes hepatocyte proliferation and inhibits apoptosis by modulating the expression of its target ACVR2B. Further, examination of the downstream Smad signalling pathway showed that ectopic expression of ACVR2B significantly promoted the phosphorylation of Smad2/3 and reversed the inhibitory effect of miR‐194 on Smad signalling (Figure 5F), indicating that miR‐194/ACVR2Bmay modulate hepatocyte proliferation and apoptosis through the Smad pathway.

## 4 DISCUSSION

The impact of liver diseases worldwide underscores the importance of elucidating the mechanisms underlying chronic liver damage and the significance of early detection for improving prevention and treatment strategies. Circulating miRNAs have emerged as biomarkers and therapeutic targets in several diseases ranging from cancer to cardiovascular diseases.^25^ In the present study, we identified miRNAs associated with CHB and focused on the role of miR‐194 in CHB‐associated liver damage. The results of miRNA microarray of samples from CHB patients and healthy controls identified miR‐194 as one of six differentially expressed miRNAs, and further analysis showed that miR‐194 was up‐regulated in correlation with the degree of liver damage in CHB patients.miR‐194 is down‐regulated in metastatic renal cell carcinoma and its overexpression suppresses liver cancer metastasis.^26^, ^27^ miR‐194 is expressed in the liver and has been suggested to play a role in hepatic stellate cell activation during fibrogenesis.^12^ The fact that miR‐194 is expressed in hepatic epithelial cells, whereas it is not detected in mesenchymal cells, suggests that it is involved in epithelial‐mesenchymal transition and plays an antimetastatic role in HCC.^27^ miR‐194 expression in the liver is modulated by transcription factor 1 or hepatocyte nuclear factor 1α (HNF1α) 28 and HNF4α,^29^ critical factors in hepatocyte development and function, and they play a role in liver tumorigenesis by targeting genes involved in tumorigenesis and tumour progression. The role of miR‐194 as a tumour suppressor in liver and gastric cancer as well as multiple myeloma was reported previously, and miR‐194 inhibits metastasis in lung cancer cells by targeting bone morphogenetic protein 1 and p27^kip1^ leading to the inactivation of TGFβ.^30^, ^31^, ^32^, ^33^

Activins, which are members of the TGFβ superfamily together with TGFβ1‐3, bone morphogenetic proteins, growth and differentiation factors, and myostatin, among others, are involved in the regulation of cell proliferation and apoptosis in the liver.^15^, ^34^ The activin receptor ACVR2B is a known target of several miRNAs including miR‐192, miR‐194, miR‐215, miR‐200c, and miR‐141 and plays an important role in the activation of activin.^35^ ACVR2B is down‐regulated in colorectal carcinoma and in poorly differentiated endometrial carcinoma, whereas it is up‐regulated in ovarian cancer cells compared with normal ovarian epithelial cells and in nephroblastoma.^35^, ^36^, ^37^, ^38^ Here, we examined the role of miR‐194 in liver regeneration mediated by the modulation of its target ACVR2B. Our results showed that miR‐194 was up‐regulated in response to PH, and the up‐regulation was correlated with hepatocyte growth. Ectopic expression of miR‐194 promoted hepatocyte proliferation and inhibited apoptosis, and this effect was abolished by ACVR2B overexpression, indicating that the effect of miR‐194 on hepatocyte growth is mediated by its target ACVR2B.

The liver is characterized by a high regenerative capacity in the presence of damaging agents.^39^ The response of the liver to injury includes the activation of non‐parenchymal cells and the production of several factors, leading to the proliferation of hepatocytes and the restoration of tissue architecture via fibrosis.^40^, ^41^, ^42^ The TGFβ signalling pathway plays an important role in liver homeostasis and in regulating hepatocyte apoptosis. Liver regeneration, which involves signalling between non‐parenchymal cells and hepatocytes, is divided into phases including the priming, proliferative, remodelling, and terminating phases, and activin A and its receptors are involved in the termination phase.^43^ ACVR2B is down‐regulated during liver regeneration after PH, and ectopic expression of ACVR2B induces apoptosis in hepatoma cells.^44^, ^45^, ^46^ Inactivating mutations of activin receptors were reported in colon, pancreatic, and prostate cancer.^47^, ^48^, ^49^ Mutations in the downstream Smad proteins have been reported in HCC^50^; therefore, we examined the involvement of Smad proteins and our results indicated that the effect of the miR‐194/ACVR2B axis on hepatocyte proliferation and liver regeneration maybe mediated by the Smad pathway.

Several miRNAs have been implicated in the process of liver regeneration. miR‐378 andmiR‐26a are down‐regulated during liver regeneration in mice exposed to PH.^51^, ^52^ miR‐26a is down‐regulated in regenerating liver tissues during the proliferative phase, suggesting that it promotes hepatocyte proliferation by regulating cell cycle progression. miR‐21 is up‐regulated during liver regeneration after PH and directly suppresses the expression of Btg2, a cell cycle inhibitor.^51^ Inhibition of miR‐21 in regenerating hepatocytes indicated that it accelerates hepatocyte proliferation by promoting cyclin D1 translation.^53^ miR‐382 is up‐regulated in the early phase of liver regeneration and promotes hepatocyte proliferation via the PTEN/Akt axis.^54^ miR‐122 is an abundant miRNA in the liver and is down‐regulated in liver cirrhosis and HCC.^55^, ^56^ miR‐122 acts as a regulator of liver fibrogenesis, negatively affecting the production of collagen by targeting prolyl 4‐hydroxylase.^57^ miR‐34a is up‐regulated during the late phase of liver regeneration and may contribute to the inhibition of hepatocyte proliferation during the termination phase by targeting inhibin βB; this suggests that it could act as a stop signal in regenerating hepatocytes similar to TGFβ and activin.^58^ Our results showing the up‐regulation of miR‐194 in correlation with hepatocyte growth during PH suggest that it may act in the early phase of liver generation by accelerating hepatocyte proliferation and inhibiting apoptosis. However, further experiments are needed to accurately determine the timing of miR‐194 expression in the regenerating liver and the phase at which it exerts its effect through the modulation of ACVR2B expression.

In summary, we showed that miR‐194 was down‐regulated in correlation with CHB‐induced liver damage. miR‐194 expression was induced during PH and promoted hepatocyte proliferation and cell cycle progression and inhibited apoptosis by down‐regulating its target ACVR2B. The present results indicate that miR‐194 promotes liver regeneration and repair through an activin/TGFβ‐related pathway. These data elucidate a potential mechanism underlying the effect of miRNAs in liver regeneration and identify the miR‐194/ACVR2B axis as a potential therapeutic target for the treatment of CHB‐related liver damage.

## CONFLICT OF INTEREST

All the authors have no conflict of interest to declare.

## AUTHOR CONTRIBUTIONS

Xue Gao performed the research and wrote the manuscript. Pan Zhao, Jie Hu and Hongguang Zhu analysed the data. Jiming Zhang and Zhongwen Zhou designed the research study. Jingmin Zhao and Feng Tang reviewed and edited the manuscript.
