# Supplementary figures and images for: MicroRNA‐194 protects against chronic hepatitis B‐related liver damage by promoting hepatocyte growth via ACVR2B
Source: J Cell Mol Med. 2018 Jul 25;22(9):4534–44. doi: 10.1111/jcmm.13714 (PMC6111826; doi:10.1111/jcmm.13714)

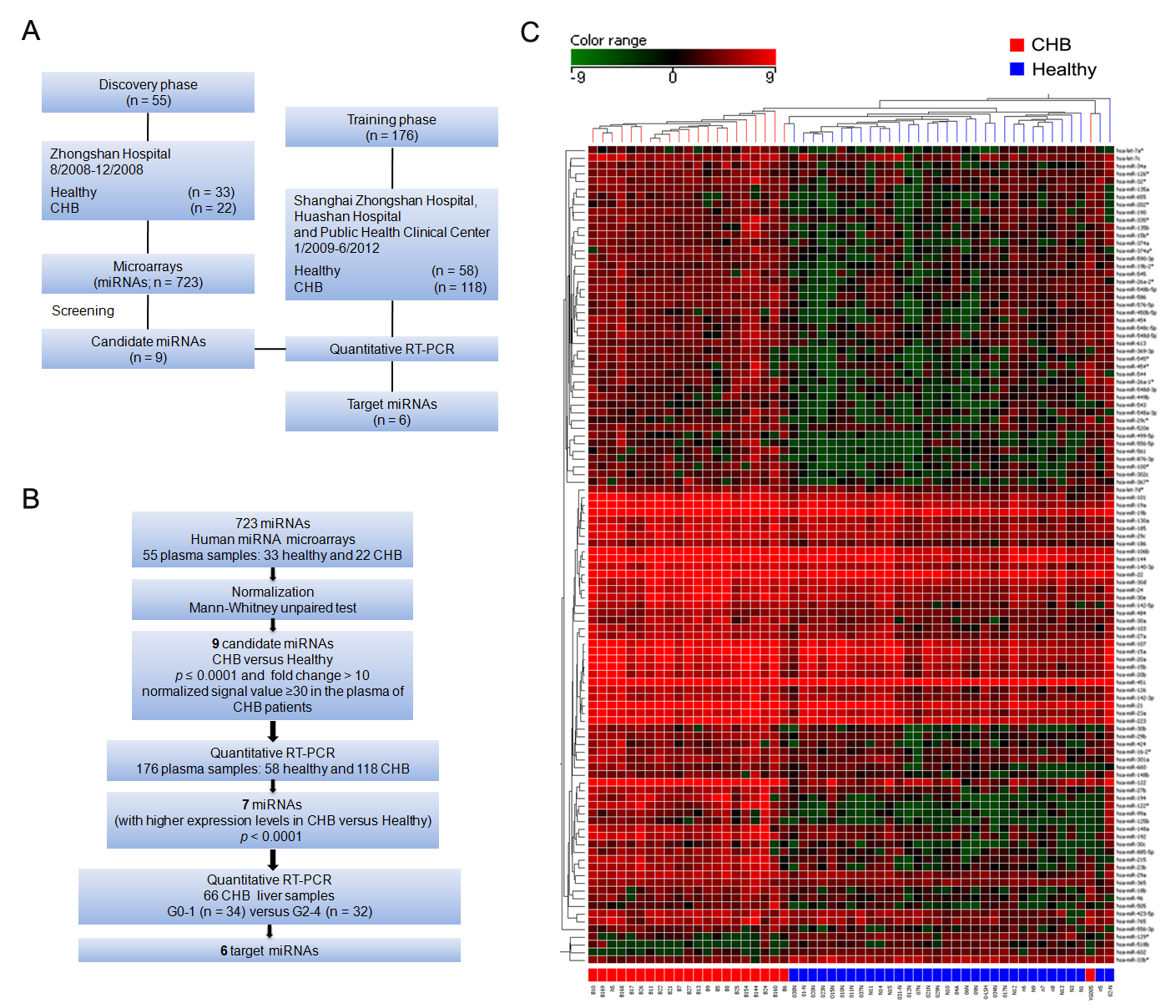

Supplement: Supplementary file 1 [file JCMM-22-4534-s001.tif]

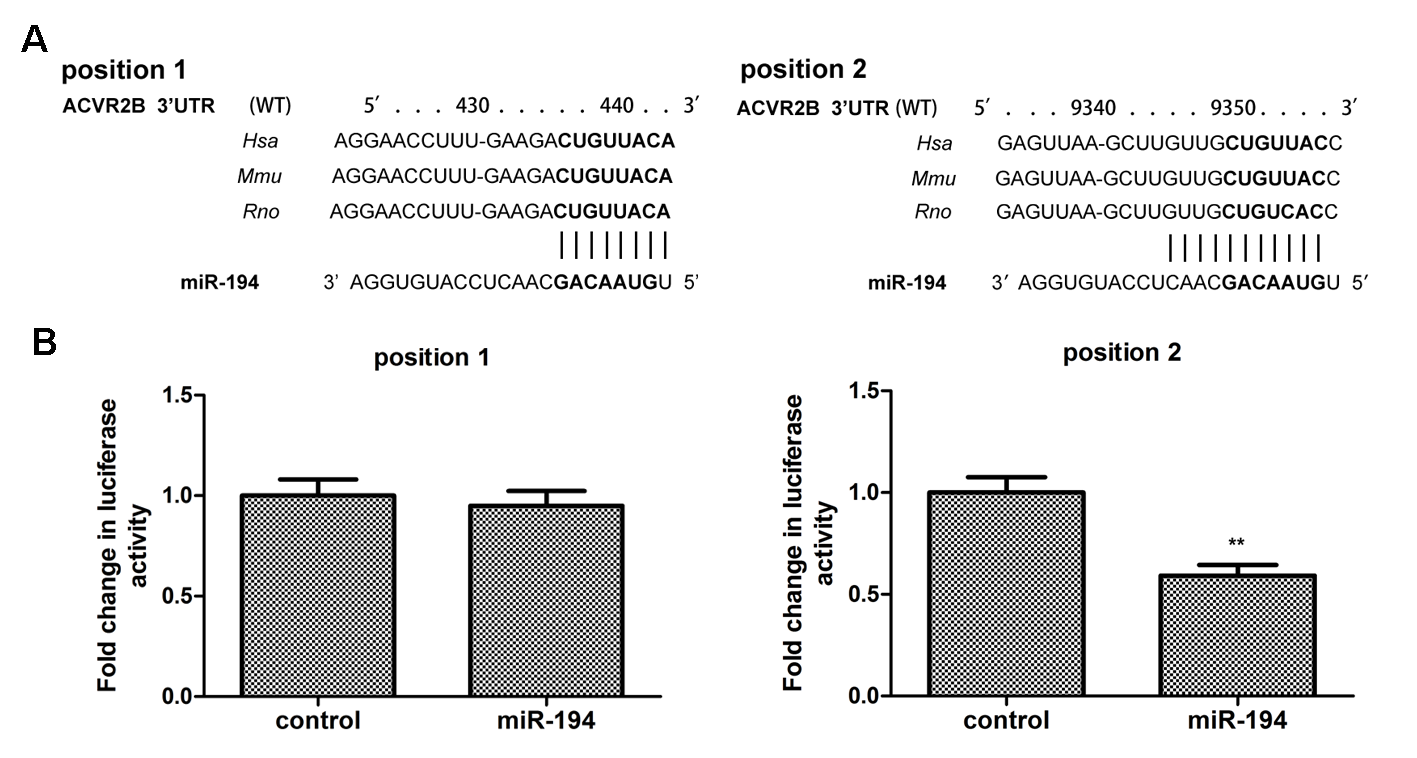

Supplement: Supplementary file 2 [file JCMM-22-4534-s002.tif]
