# Supplementary material for: MicroRNA‐194 protects against chronic hepatitis B‐related liver damage by promoting hepatocyte growth via ACVR2B
Source: J Cell Mol Med. 2018 Jul 25;22(9):4534–44. doi: 10.1111/jcmm.13714 (PMC6111826; doi:10.1111/jcmm.13714)
Supplement: Supplementary file 3 [file JCMM-22-4534-s003.doc]

| **Table S1 Eligibility criteria for selection of the study subjects** |
| --- |
| 1. Age ≥18 years and ≤90 years |
| 2. Not currently residing in an institution, such as a prison, nursing home, or shelter |
| 3. Not severely ill in the intensive care unit |
| 4. With the capability to give informed consent |
| 5. Encountered between August 2008 and June 2010 |
| **Healthy individuals** |
| 1. Had the medical check-up in Zhongshan Hospital |
| 2. In healthy condition without malignancy and system infection (lung, gastrointestinal tract, urinary tract) |
| **Chronic hepatitis B (CHB) patients** |
| 1. HBsAg-positive >6 months |
| 2. Serum HBV DNA >20,000 IU/mL (10^5^copies/mL), lower values 2,000-20,000 IU/mL (10^4^-10^5^ copies/mL) are often seen in HBeAg-negative chronic hepatitis B |
| 3. Persistent or intermittent elevation in ALT/AST levels |
| 4. All the patients had full pathologic data from liver biopsy |
