# Supplementary material for: MicroRNA‐194 protects against chronic hepatitis B‐related liver damage by promoting hepatocyte growth via ACVR2B
Source: J Cell Mol Med. 2018 Jul 25;22(9):4534–44. doi: 10.1111/jcmm.13714 (PMC6111826; doi:10.1111/jcmm.13714)
Supplement: Supplementary file 4 [file JCMM-22-4534-s004.doc]

| **Table S2. Clinical Characteristics of the Study Subjects** | | | | |  |
| --- | --- | --- | --- | --- | --- |
| **Variable** |  | **Plasma** | | **Tissue** | |
|  |  | **Microarray** | **qRT-PCR** | **qRT-PCR** | |
| **Healthy** count (%) |  | **33** | **58** |  | |
| Age (mean ± SD) |  | 43 ± 14 | 43±13 |  | |
| Sex | Male | 13 (39) | 31 (53) |  | |
|  | Female | 20 (61) | 27 (47) |  | |
| ALT | ≤ 40 U/L | 25 (76) | 52 (90) |  | |
|  | > 40 U/L | 3 ( 9) | 6 (10) |  | |
|  | Missing | 5 (15) | 0 (0) |  | |
|  | | | | | |
| **CHB** count (%) |  | **22** | **118** | **66** | |
| Age (mean ± SD) |  | 42±11 | 38±11 | 37±10 | |
| Sex | Male | 18 (82) | 80(68) | 55 (83) | |
|  | Female | 3 (13) | 38(32) | 11(17) | |
| ALT | ≤ 40 U/L | 5 (23) | 40(34) | 20 (32) | |
|  | > 40 U/L | 15 (68) | 78(66) | 42 (68) | |
|  | Missing | 1 ( 5) | 0 ( 0) | 4 ( 6) | |
|  | | | | | |
| Scheuer classification (liver biopsy) | | | | | |
| Necrosis/inflammation | Grade 0 |  | 28 (24) | 17 (26) | |
|  | Grade 1 |  | 28 (24) | 17 (26) | |
|  | Grade 2 |  | 32 (27) | 14 (21) | |
|  | Grade 3 |  | 30 (25) | 18 (27) | |
|  | Grade 4 |  | 0 (0) | 0 (0) | |
