# Supplementary material for: MicroRNA‐194 protects against chronic hepatitis B‐related liver damage by promoting hepatocyte growth via ACVR2B
Source: J Cell Mol Med. 2018 Jul 25;22(9):4534–44. doi: 10.1111/jcmm.13714 (PMC6111826; doi:10.1111/jcmm.13714)
Supplement: Supplementary file 5 [file JCMM-22-4534-s005.doc]

**Table S3** Expression profiles of candidate miRNAs between healthy individuals and CHB patients

|  | **Microarray** | | | |  | **qRT-PCR** | | | | | | |
| --- | --- | --- | --- | --- | --- | --- | --- | --- | --- | --- | --- | --- |
| miRNA | P value | Mean signal | | Fold change  CHB/healthy | | |  | P value | Relative expression | | | Fold change  CHB/healthy |
|  |  | Healthy  (n=33) | CHB  (n=22) |  |  |  |  |  | Healthy  (n=58) | CHB  (n=118) | |  |
| hsa-miR-122* | <0.0001 | 0 | 53 | 351 | | |  | ND | ND | ND | | ND |
| hsa-miR-194 | <0.0001 | 0 | 41 | 97 | | |  | 0.0001 | 0.2 | 1.1 | | 4.4 |
| hsa-miR-23b | <0.0001 | 1 | 50 | 48 | | |  | ND | ND | ND | | ND |
| hsa-miR-215 | <0.0001 | 2 | 58 | 34 | | |  | <0.0001 | 0.1 | 1.1 | | 20 |
| hsa-miR-122 | <0.0001 | 36 | 1126 | 32 | | |  | <0.0001 | 5.2 | 104 | | 20 |
| hsa-miR-148a | <0.0001 | 3 | 55 | 21 | | |  | <0.0001 | 0.9 | 2.5 | | 2.7 |
| hsa-miR-27b | <0.0001 | 4 | 55 | 16 | | |  | <0.0001 | 0.3 | 2.4 | | 7.4 |
| hsa-miR-192 | <0.0001 | 6 | 82 | 14 | | |  | <0.0001 | 2.4 | 15 | | 6.1 |
| hsa-miR-29b | <0.0001 | 3 | 36 | 14 | | |  | <0.0001 | 0.0 | 0.2 | | 5.2 |
| ND, not determined, the microRNA did not pass the quality control. | | | | | | | | | | |  |  |
