# Supplementary material for: MicroRNA‐194 protects against chronic hepatitis B‐related liver damage by promoting hepatocyte growth via ACVR2B
Source: J Cell Mol Med. 2018 Jul 25;22(9):4534–44. doi: 10.1111/jcmm.13714 (PMC6111826; doi:10.1111/jcmm.13714)
Supplement: Supplementary file 6 [file JCMM-22-4534-s006.docx]

**Appendix S1**

**Plasma preparation and RNA isolation**

For plasma preparation, peripheral blood (4 ml) was drawn into EDTA tubes. Within 2 h, the tubes were subjected to centrifugation at 820 g for 10 min. Then, 1 ml aliquots of the plasma were transferred to 1.5 ml tubes and centrifuged at 16,000g for 10 min to pellet any remaining cellular debris. Subsequently, the supernatant was transferred to fresh tubes and stored at –80°C.

For the plasma samples, total RNA was extracted using a mirVana PARIS miRNA Isolation kit according to the instructions from the manufacturer (Ambion, Austin, TX, USA). The concentration was quantified by NanoDrop 1000 Spectrophotometer (NanoDrop Technologies, Waltham, MA, USA).

**Microarray hybridization**

Human miRNA microarrays (Agilent Technologies, Santa Clara, CA, USA) were used to identify candidate miRNAs in plasma samples. The microarray contains probes for 723 human miRNAs from Sanger database v.10.1. The labelling and hybridization were performed according to the protocols in the Agilent miRNA microarray system. The raw signals obtained for single-colour CY3 hybridization were normalized by a stable endogenous control, miR-1228. Then, a log transform with base 2 was performed. Samples showing intra-array coefficients of variation (CV) across replicated spots on an array of >15% or positive signals of <5% were considered unreliable and excluded from further analysis. A detectable miRNA was defined as the miRNA with positive signals on microarrays in >50% of plasma samples from any one of the two category subjects.
